# A Sober Critic of Koch

**Published:** 1890-12-27

**Authors:** 


The Hospital

A Weekly Journal of

Science, Medicine, Nursing, and Philanthropy.

*For Hospitals, Asylums, Infirmaries, Dispensaries, Medical Practitioners, Students, Nurses, and the Charitable Public.*

Vol. IX.—No. 222.] [December 27, 1890.

### A Sober Critic of Koch.

The general excitement about Dr. Koch's cure for consumption has died down. There need be neither surprise nor regret felt that it should have been so great and so universal. Consumption is a great and universal scourge, and if it could be banished from the face of the earth, its disappearance would mark an epoch in the progressive history of the human race. Dr. Theodore Williams has visited Berlin, and investigated Koch's methods and results on the spot. There is no higher authority on consumption, in all its forms, in the world than Dr. Theodore Williams. Readers, whether medical or lay, will do well to attach much importance to his statements. In the treatment of lupus, Dr. Theodore Williams holds that there are results to be seen at Berlin which are admirable. What Dr. Williams calls "strumous" bones and joints, but what are called by some other pathologists "tubercular" bones and joints, are stated also to have been strikingly improved by a succession of Koch's injections. On this point Dr. Williams says that "the changes in the children in Professor von Bergman's and Dr. Levy's clinics impressed me greatly."

But it is phthisis of the lungs, or consumption, that Dr. Koch has announced to the world the discovery of a cure for. The doctors who have flocked to Berlin have flocked to see consumption cured; the patients who have hurried thither with pathetic haste have been consumptive patients. Dr. Theodore Williams is a specialist in consumption, and it is on this, the most important aspect of Koch's discovery, that he is specially entitled to be heard. Is Dr. Koch's lymph injection a cure for consumption or is it not? To this one point Dr. Theodore Williams has directed all his inquiries; and on this one point those who wish for information which is worthy of being called scientific will do well to concentrate their intelligence as they read the results of his experience. The consumptive patients at Berlin at the present moment are very numerous. Dr. Williams visited and carefully examined about a hundred of them in their beds in the different wards. Among the hundred were patients at almost every stage of the disease: some had been phthisical for months, others for years; some had lung cavities so small as to be scarcely discoverable, others had a degree of destruction of lung tissue which rendered their recovery hopeless. The mass of the cases were such as may ordinarily be found at the Brompton Hospital for Consumption, or at any of the other chest hospitals —"partly cases of tuberculisation and partly cavity cases."

It is important to consider two things at this point. First, how long the majority of the hundred cases seen by Dr. Williams had been under treatment; and, secondly, how long theoretically does a typical case of moderately advanced phthisis require for its cure by Dr. Koch's method. But from a practical point of view we have also to ask, What is the nature of the cure which satisfies the doctor? and what the nature of the cure which satisfies, and alone should satisfy, the patient and his friends? With regard to the first point, most of the cases seen by Dr. Theodore Williams had been under treatment from two to four weeks; a few of them, however, had been treated a good deal longer. With regard to the second, there seems to be no general average of duration of treatment established, and indeed it is difficult to see how this could be. But on the third point, a case is considered by Dr. Koch and his friends to be cured when the injection of the lymph fails to produce any reaction. That many patients who no longer react to injection are in no sense "cured," as a layman would understand the expression, is only too evident from Dr. Theodore Williams' published observations.

"Throat consumption" offers special advantages to the experimenter, because by means of the laryngoscope he can see with his own eyes the results produced by each injection of the lymph. Dr. Theodore Williams describes the effects of the injections in these cases as very striking. At the seat of the disease the laryngeal mucous membrane swelled up, and greyish sloughs appeared, and were separated from the deeper parts under the observer's eyes. "In these cases," says Dr. Williams, "improvement is common, but complete cure rare." The questions to be asked are, Does complete cure ev[illegible] take place? And, if so, how long does it last?

Ordinary cases of consumption of the lungs do no[illegible] offer the same facilities for observation as laryngeal cases. It is the custom to treat patients of this class with successive injections of the lymph, until a period is reached when no reaction follows. The patient is then said to be cured. Is he cured? Dr. Theodore Williams makes some practical observations on this fundamental question. He says, "Cases occur which cease to react under strong injections, but still present the physical signs of phthisis, and whose sputum contains tubercle bacilli. . . . I examined in one of the clinics a middle-aged man, with a well-marked cavity in the upper lobe of the right lung of some standing, with marked fibrosis of the rest of the lung, and with some necrosis of the sternum, the supervention of which had relieved the cough, expectoration, and other lung symptoms. The sputum contained no tubercle bacilli. This patient gave no reaction after strong injections, but I should be sorry to affirm that he was not tubercular." No one, it seems to us, who has not determined beforehand to find cures, would consider a case of this kind cured. For our part, whatever be the theories of ardent bacteriologists, we do not hold a patient to be cured at all unless he is restored to health.

Dr. Theodore Williams seems to hint at the terrible possibility of the setting up of tuberculosis where it did not exist before, by means of Koch's injections; and this is merely one of the dangers which will strike every man of any clinical experience. He records the case of a young man who was suffering from a cough and expectoration. This young man was injected with Koch's lymph, but the injection was not followed by any kind of reaction. Now, according to the theory, if there be any tuberculosis present an injection of the lymph is bound to produce a reaction. If no reaction follows it is proof positive that there is no tuberculosis about the patient. A second time the young man was injected, and after the interval of a few days. But still there was no reaction. According to the theory the absence of tuberculosis was thus demonstrated by proof upon proof. But a third and stronger injection was used, and then an unmistakeable reaction ensued; the temperature rose to 102·2° F.; crepitations were heard at the apex of one lung; and tubercle bacilli were detected in the sputum. Now, as we have said, if two injections were followed by no reaction, then, according to the theory, no tuberculosis could possibly be present. But if a third injection is followed by a reaction, then the presumption is that tuberculosis has developed since the date of the second injection; and what is so likely as that the injection itself has been the cause of the development of the tuberculosis? Dr. Williams had the advantage of being present at the post-mortem examination of two persons who had died in the Charité Hospital after having received several injections of the lymph. He says of these cases that practically the diseased lungs were in exactly the same condition as are the diseased lungs of persons who die of phthisis, after being treated in the ordinary way. This, as every physician knows, is a fact of which the significance can scarcely be exaggerated.

In a word, whilst some cases seem to improve under Dr. Koch's treatment, others as certainly die. The great majority appear to lose weight rather than to gain it: weight is rarely gained. Of the 100 cases examined by Dr. Theodore Williams he could only find eight of whom he could say with certainty that they were genuinely and definitely improved. Of these, he asserts that the improvement was not greater than would have been made by similar patients at the Brompton or any other consumption hospital, and he significantly adds that the successes obtained by Dr. Koch and his assistants "would not for an instant compare with the results obtained at the high altitude sanatoria, such as Davos, St. Moritz, or Colorado, where the restoration to health is often complete, and no physical signs or tubercle bacilli remain." Readers may take it that the candid judgment of Dr. Theodore Williams is worth more than whole ship-loads of daily newspaper reports; and is decidedly to be preferred to the necessarily partial conclusions of Dr. Koch and his German and foreign admirers. We repeat what we have so often said before, that the present is the testing period; the day of proved results has yet to dawn.

